# ‘On Your Feet to Earn Your Seat’, a habit-based intervention to reduce sedentary behaviour in older adults: study protocol for a randomized controlled trial

**DOI:** 10.1186/1745-6215-15-368

**Published:** 2014-09-20

**Authors:** Benjamin Gardner, Ingela Thuné-Boyle, Steve Iliffe, Kenneth R Fox, Barbara J Jefferis, Mark Hamer, Nick Tyler, Jane Wardle

**Affiliations:** Health Behaviour Research Centre, Department of Epidemiology and Public Health, University College London, London, UK; Research Department of Primary Care & Population Health, University College London, London, UK; Centre for Exercise, Nutrition and Health Sciences, University of Bristol, Bristol, UK; Research Department of Primary Care & Population Health, Population Health Domain Physical Activity Research Group, University College London, London, UK; Population Health Domain Physical Activity Research Group, Department of Epidemiology and Public Health, University College London, London, UK; Department of Civil, Environmental and Geomatic Engineering, University College London, Gower Street, London, WC1E 6BT UK

**Keywords:** Sedentary behaviour, physical activity, behaviour change, habit, older adults

## Abstract

**Background:**

Many older adults are both highly sedentary (that is, spend considerable amounts of time sitting) and physically inactive (that is, do little physical activity). This protocol describes an exploratory trial of a theory-based behaviour change intervention in the form of a booklet outlining simple activities (‘tips’) designed both to reduce sedentary behaviour and to increase physical activity in older adults. The intervention is based on the ‘habit formation’ model, which proposes that consistent repetition leads to behaviour becoming automatic, sustaining activity gains over time.

**Methods:**

The intervention is being developed iteratively, in line with Medical Research Council complex intervention guidelines. Selection of activity tips was informed by semi-structured interviews and focus groups with older adults, and input from a multidisciplinary expert panel. An ongoing preliminary field test of acceptability among 25 older adults will inform further refinement. An exploratory randomized controlled trial will be conducted within a primary care setting, comparing the tips booklet with a control fact sheet. Retired, inactive and sedentary adults (*n* = 120) aged 60 to 74 years, with no physical impairments precluding light physical activity, will be recruited from general practices in north London, UK. The primary outcomes are recruitment and attrition rates. Secondary outcomes are changes in behaviour, habit, health and wellbeing over 12 weeks.

**Discussion:**

Data will be used to inform study procedures for a future, larger-scale definitive randomized controlled trial.

**Trial registration:**

Current Controlled Trials ISRCTN47901994.

**Electronic supplementary material:**

The online version of this article (doi:10.1186/1745-6215-15-368) contains supplementary material, which is available to authorized users.

## Background

Sedentary behaviour, which has been defined as ‘any waking behaviour characterized by an energy expenditure ≤1.5 metabolic equivalents while in a sitting or reclining posture’ [1], represents a risk factor for adverse physical and mental health outcomes [2, 3], independently of physical activity [4, 5]. Of all age groups, older adults tend to spend most time in sedentary activity and least time in physical activity [6].

Behaviour change interventions are needed to replace sedentary behaviour with physical activity, cost-effectively, in older adults. Four types of physical activity are recommended in older adulthood: aerobic, muscle-strengthening, flexibility, and balance exercises [7, 8]. There is increasing evidence that standing, a light-intensity balance activity, yields cardiometabolic health benefits relative to sitting [9]. The benefits of reducing sedentary behaviour in older adults should therefore be twofold, reducing the risks associated with prolonged sitting, and achieving the benefits associated with physical activity. While decline in physical functioning can impose limits on activity in older adults, light-intensity physical activity is usually feasible, and can increase fitness, reduce morbidity and mortality, and promote natural uptake of more intensive activities [10–14].

Many interventions have sought to increase physical activity among older adults [15–17], but few have explicitly targeted sedentary behaviour [18–20]. Two studies have shown that provision of individualized consultations and personalized accelerometer feedback is associated with reductions in sitting time and increases in light and moderate-to-vigorous physical activity in older adults [19, 20]. Although these were uncontrolled pilot studies, with relatively small samples (*n* ≤ 69) and short-term follow-up (≤24 days), the results support the potential for interventions to primarily reduce sedentary behaviour and, in so doing, achieving the secondary aim of increasing physical activity.

Many behaviour change interventions have short-term effects, which are eroded when the intervention is withdrawn [21]. This can reflect a progressive decline in motivation, or a dependence on external support [22]. The ideal sedentary behaviour reduction intervention would be self-sustaining. Behavioural psychology suggests that forming ‘habits’ can ‘lock in’ behaviour gains over time. ‘Habitual behaviours’ are actions that are driven by impulses that are activated by encountering a situation (environmental cue) in which the action has been repeatedly performed in the past [23]. The environmental cue could be, for example, a geographical location, a person or the previous action within an existing routine [24–26]. Context-consistent repetition reinforces a mental cue-behaviour association [27], so that an impulse to do the action is activated when the cue is encountered [23]. While motivation and effort are required to initiate and sustain the early stages of a habit formation attempt, as the habit forms, control over behaviour is delegated to the external cue, and less conscious effort is required to do the action [28], so that behaviour is sustained even when intentions are weak [29]. Habitual behaviours should continue to occur as frequently as the cue is encountered [30, 31], potentially becoming self-perpetuating. Habit formation thereby offers a potential means to maintain gains from behavioural interventions after withdrawal of external support [30].

### Aims and objectives

This protocol describes work to develop a theory- and evidence-based behaviour change intervention aiming to displace prolonged sitting time (sedentary behaviour) with at-least-light-intensity physical activity in older adults. The intervention seeks to promote the formation and integration of physical activity habits into existing normally sedentary routines. An exploratory randomized controlled trial (RCT) will compare the intervention with a control fact sheet setting out national guidelines for physical activity and sedentary behaviour. The primary objective of the RCT is to assess the feasibility of our intervention and our study procedures, as a basis for informing a large-scale future RCT. The secondary objective is to assess changes in behaviour, health and wellbeing. The intervention is being developed in line with the Medical Research Council complex interventions framework [32]. This protocol has been prepared in accordance with Standard Protocol Items: Recommendations for Interventional Trials (SPIRIT) and Template for Intervention Description and Replication (TIDieR) guidelines [33, 34], completed checklists for which are provided as additional files (see Additional files 1 and 2).

## Methods

### Theoretical basis: the ‘habit formation’ model

Behaviour change interventions explicitly based on theory tend to be more effective [35, 36]. The present intervention is based on the ‘habit formation’ model [25, 37]. Habit typically forms asymptotically, with initial repetitions causing greatest gains in automaticity (that is, habit strength), and the contribution of further repetitions reducing until a plateau is reached [25]. Habit formation requires motivational and volitional skills and resources to initiate and repeat behaviour until it becomes automatic [38]. Repetition in the early stages is best facilitated where the individual is intrinsically motivated (that is, pursues behaviour for its personal value, rather than to satisfy external demands) [39, 40], where target behaviours are manageable and realistic [41], and where people actively monitor their progress [37]. This can sustain interest through the effortful stages of the habit formation process [42]. Simpler behaviours may become habitual more quickly than more complex actions [25], and so a ‘small changes’ approach, based on minor modifications to existing practices, may be more suited to habit formation than the pursuit of major behaviour change [37].

The few behaviour change interventions that have applied these principles have found promising results [19, 43–46]. For example, the ‘Ten Top Tips’ weight loss intervention centred on a leaflet outlining ten simple recommendations (‘tips’) designed to create healthy habits (for example, ‘try to eat at roughly the same time each day’) [44]. An exploratory trial among overweight and obese participants found that intervention recipients lost more weight than a matched waiting-list control over 8 weeks, and maintained weight loss 6 months after active treatment ceased [44]. Habit gains correlated with weight loss, and participants reported that adherence to the tips became ‘second nature’, suggesting that habit formation underpinned intervention effects [44, 47].

### Preliminary work to generate intervention content

This intervention was modelled on the ‘Ten Top Tips’ intervention [44]. The aim was to develop a booklet presenting ten simple physical activity tips selected to be conducive to habit formation, with accompanying motivational text on the importance of reducing sedentary behaviour and increasing physical activity in later life. Empirical evidence was gathered to inform intervention content within the habit formation framework, in four stages; a supplementary figure presents an overview of this process (see Additional file 3). First, a group of older adults discussed attitudes, barriers and facilitators of sedentary behaviour and physical activity in focus groups and individual interviews. This information informed the generation of physical activity tips and accompanying motivational text. Second, expert panel feedback informed a second iteration of intervention content. Third, an additional focus group session and individual interviews among the older adult panel informed a further iteration. Finally, an independent group of 23 older adults rated the intervention content for readability, difficulty, motivation and performance likelihood, informing further refinement. This preliminary work was approved by the University College London ethics committee (1427/003, 4025/001).

### First round of focus group and interviews

A panel of older adults convened by Age UK took part in a focus group session (*n* = 10) and individual interviews (*n* = 17). Participants were self-reportedly 60 to 75 years, retirees, largely inactive (<30 minutes of leisure time physical activity of at least three metabolic equivalents per week) and sedentary (>6 leisure time hours spent sitting per day), motivated to reduce their inactivity and physically capable of light physical activity. Topics included experiences of and attitudes towards sedentary behaviour and physical activity, routines and frequently encountered contexts and existing sedentary behaviour habits. Participants were invited to brainstorm ideas for incremental increases in physical activity feasible for repetition by older adults. Thematic analysis identified six themes.

#### Types of activity

Participants found it difficult to identify explicit activities suitable for the booklet. However, discussions indicated that mundane everyday activities (for example, stair use, walking, housework, stretching and strengthening exercises) would be feasible and acceptable.

#### Context

The context for recommended physical activity was seen as important for repetition and motivation; for example, indoor activities would permit year-round activity by avoiding reliance on the weather. Participants felt that physical activity opportunities within existing inactive routines, for example, while watching television at home, or when waiting at a bus stop, should be exploited. Many felt that recommending social forms of physical activity (for example, walking with friends) could bolster motivation.

#### Nature of physical activity

Participants stressed that recommended physical activity should be achievable for older adults, and not ‘hard work’. Small changes, based on starting with minimal-intensity physical activity and experimenting with ways to increase physical activity within the constraints of physical capability and health status, were preferred.

#### Motivators

Many participants said that recommended physical activities should be interesting, safe and pleasurable, and yield noticeable improvements in mood, confidence and energy, while reducing pain.

#### Barriers

Exercise feeling like a ‘chore’ or a ‘big deal’ were common barriers to physical activity. Structured or ‘artificial’ activities, such as going to the gym or using an exercise bicycle, had negative connotations. Other barriers included health status, especially arthritis, pain, swelling and living with multiple conditions and fear of falling.

#### Booklet design

Participants advised that the intervention booklet should include simple explanatory illustrations and that text should be motivational, pitched towards older adults and in large print. They recommended avoiding the term ‘exercise’, which for some had negative connotations stemming from unpleasant experiences of physical education at school.

Synthesis of these data led to the generation of 18 physical activity-increasing and sedentary behaviour-reducing tips (incorporating aerobic, muscle-strengthening, flexibility and balance exercises), and brief motivational texts outlining the importance of reducing sitting and increasing physical activity in older adulthood, and explaining the habit formation approach. Text and tips were worded in autonomy-supportive language, to foster intrinsic motivation [48].

### Expert panel

A multidisciplinary expert panel was convened to provide feedback on the tips and text. A total of 32 eligible experts, known to the research team, or known to have done relevant work, were invited, of whom 15 agreed to participate, representing sports and exercise science (*n* = 5), general practice (*n* = 2), physiotherapy (*n* = 1), health psychology (*n* = 2), geriatrics (*n* = 2), ageing (*n* = 2) and physiology (*n* = 1). The experts were invited to comment on the selection, intensity and safety of the tips, and their motivational quality more broadly, and to suggest alternative content. The text was subsequently refined, and the number of tips reduced from 18 to 16.

### Second round of focus group and interviews

Next, a second focus group session (*n* = 6) and individual interviews (*n* = 11) with the older adult panel canvassed views on the revised intervention content. Discussions addressed feasibility, comprehension, barriers to adherence and suggestions for improvement. These insights informed further refinement of intervention content, reducing the number of tips to ten, which retained coverage of the four physical activity sub-types (aerobic, muscle strengthening, flexibility, balance).

### Nominal group ratings

A postal survey was then conducted, to assess acceptability among an independent sample of inactive and sedentary retired older adults identified and recruited by Age UK. Participants were asked to rate the readability, difficulty, motivation and performance likelihood of each of the ten tips, on a scale of 1 to 4 (1, least positive; 4, most positive), and provide optional free-text feedback. Of 40 surveys distributed, 23 (57.5%) were returned. The tips were judged to be readable (grand mean, 3.4), easy (3.0), motivating (2.8) and likely to be performed (3.0). The motivational text was rated as interesting (3.4) and easy to understand (3.4). A graphic designer was then employed to create a visually attractive intervention booklet, with photos added to clarify some tips.

### Preliminary field testing

The acceptability of the intervention booklet is currently being tested among 25 retired, sedentary and inactive adults aged 60 years and over, living in sheltered accommodation in London. Participants will be guided through the intervention booklet and asked to use it, along with eight 7-day ‘tick sheets’ to self-monitor adherence to the tips, for eight weeks. Prior to intervention administration, and at 4- and 8-weeks follow-up, participants will complete quantitative measures of habit strength (that is, automaticity of behaviours), levels of sedentary behaviour and physical activity, health and wellbeing. Semistructured interviews at 12-week follow-up will address experiences of using the booklet. We will also examine the feasibility of various outcome measures. Results will inform further refinement of the intervention, as necessary.

### Exploratory trial

#### Study design

An exploratory two-arm pilot RCT will be undertaken to evaluate the intervention. Participants will be randomized to receive either the habit-based booklet (intervention condition), or an existing UK National Health Service (NHS) fact sheet on physical activity and sedentary behaviour in older adulthood (control condition). Assessments will be made at baseline, and 8 and 12-weeks post-baseline (follow-up). Participants will be blinded to condition, but owing to staffing constraints, the researcher administering the intervention will be required to obtain outcome measures, so will be unblinded. Primary outcome measures will be recruitment and attrition rates, and secondary outcome measures will be changes in behaviour, habit, health and wellbeing. The study was registered on the International Standard RCT Number (ISRCTN) Register^a^ (ISRCTN47901994) in January 2014. The trial start date differs from that stated on the ISRCTN Register (April 2014) because of multiple unforeseen delays. The new planned start date is September 2014, and the trial is scheduled to end in February 2015. Recruitment had not begun at the time of submission of this manuscript.

#### Study setting

Participants (*n* = 120) will be identified through general practices in Barnet and Enfield, boroughs in north London, UK, chosen because of the high percentage of older adults (≈15%). The sample size has been pragmatically determined, to obtain sufficient data to capture variability and inform a power calculation for a larger-scale definitive RCT, while minimizing trial costs [49]. Consent will be obtained at the general practice, and data collection and treatment administration undertaken in participants’ homes.

#### Inclusion and exclusion criteria: practices

Only practices with a private space available for consenting participants will be recruited. Practices that have recruited patients to a physical activity promotion or sedentary behaviour reduction study in the previous 3 months will be excluded.

##### Inclusion criteria: patients

Participants will be aged 60 to 74 years, retired, inactive (≤30 consecutive minutes of leisure time physical activity of ≥3 metabolic equivalents per week) and sedentary (≥6 total leisure time hours sitting per day).

##### Exclusion criteria: patients

Anyone who reports a disabling impairment that prevents them from engaging in regular light-intensity physical activity, or has participated in the previous 3 months in a physical activity promotion or sedentary behaviour reduction study, will be excluded. Patients without the mental capacity to provide informed consent will also be excluded. Limited funding precludes translation of study materials or the use of interpreters, so anyone unable to read or write English will be ineligible. To avoid contamination, people in the same household as another person recruited to the study will not be eligible.

#### Recruitment procedure: practices

Practices will be selected to maximize the chances of recruiting patients from a variety of ethnicities and socioeconomic backgrounds. Practices identified by the North Central London Research Consortium will be approached via an emailed study advertisement. We intend to recruit from seven general practices at most. One practice from each borough will be engaged during the first month of recruitment, so that study procedures may be piloted prior to the engagement of multiple practices.

#### Recruitment procedure: patients

**Figure 1:**
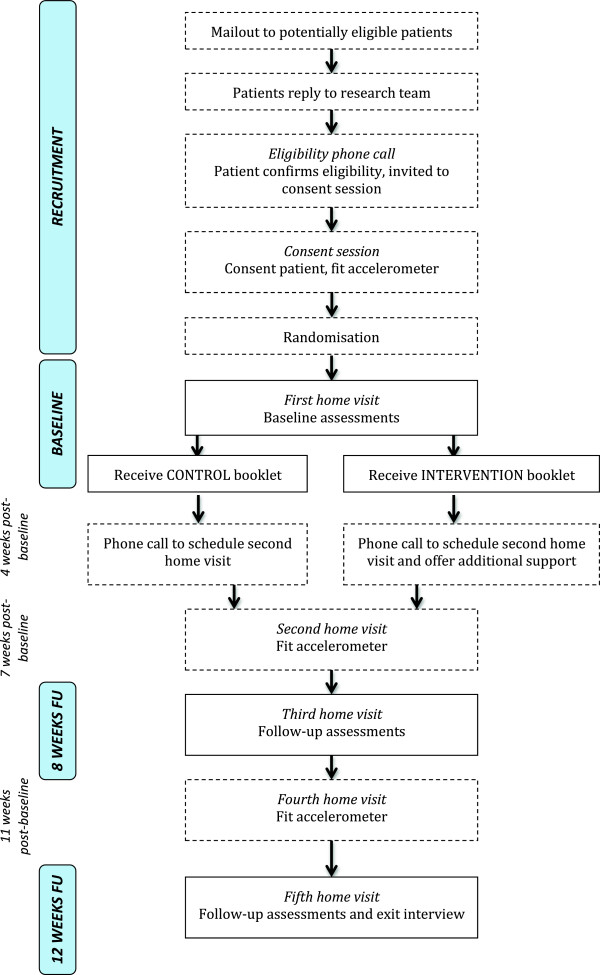
**Participant flow.**

Figure 1 illustrates the participant flow through the study. In each practice, a computer search of electronic patient record databases will be run by a practice administrator to identify potentially eligible participants. That is, patient records will indicate that they are: aged 60 to 74 years; ‘inactive’ or ‘moderately inactive’ (according to data collected by practices in 2013 using the General Practice Physical Activity Questionnaire [50], administered to all NHS patients aged 60 years or above); not in employment; not so physically impaired as to preclude light physical activity (that is, patients with codes ‘mobility impaired’, ‘wheelchair user’, ‘lower limb amputee’ or ‘housebound’ will be excluded); English-speaking; and able to give fully informed consent (that is, patients with codes denoting mental health problems, palliative care, or a learning disability will be excluded).

Study information packs will be sent by post by the practice to a random sample of at most 300 eligible patients, with additional mail outs conducted if response rates among the first 300 are low (below 20%). The study pack will contain a study invitation letter, information sheet and business reply envelope. All documents within the pack will feature unique codes that identify the practice and recipient number, permitting calculation of response rates. Both the invitation letter and information sheet will list eligibility criteria for consideration for entry into the study. A tear-off sheet for return to the research team will be provided, so that interested patients can self-report their eligibility, contact details and consent to be contacted further by the research team.

Those who return the tear-off sheet will be phoned by a research team member, and asked to confirm their age and retirement status. Patients’ physical capability will be assessed by asking them to confirm that they could comfortably participate for at least 10 consecutive minutes in a set of physical daily activities, chosen to cover stretching, strengthening, balance and aerobic activities, each of which feature in the intervention booklet (for example, walking, housework). Patients will self-report their physical activity and sedentary behaviour over the telephone, using an adapted and validated short form of the International Physical Activity Questionnaire, which documents daily activity and sitting [51], and measures of time spent in specific sitting-based activities [52]. Patients confirmed as eligible will be invited to an appointment with a researcher at the general practice, at which they will provide written consent. Patients will be asked to bring their invitation letter to this session, to allow recording of per practice recruitment rates. A baseline measurement and treatment administration session will be arranged to take place a week later at the participant’s home. Recruitment to baseline is due to cease in December 2014, owing to time constraints.

### Consent

Patients will be advised of their right to decline participation, and to withdraw at any time without giving reasons. To allow for analysis of attrition, participants will be informed that, if they do not complete the study, their data will remain within the study for the purposes of follow-up and data analysis, unless they tell us to withdraw their data.

### Estimated recruitment rates and study duration

Total patient populations of the target practices vary from 4,500 to 14,000. Based on UK census data, we estimate that 10% of all registered patients at these practices will be aged 60 to 74 years [53]. Data from the 2008 Health Survey for England indicate that around half of all people aged 65 to 74 years, and 35 to 40% of those aged 55 to 64 years, self-report an average of 6 or more daily hours of sedentary behaviour [6]. We therefore estimate that 40% of older adults in each practice (4% of all patients) will meet our inactivity and sedentary criteria. We have based our estimates on self-reported data, as these are likely to be conservative; accelerometry data suggest that sedentary time may average 10 to 11 hours per day among adults aged 70 years or above [54]. We estimate a response rate of 30%, and so expect 1% of all patients in each practice to participate, but have allowed for recruitment rates lower than this by using up to seven practices.

### Randomization

Participants will be block randomized to study conditions, using a 1:1 allocation ratio, according to a random number list, computer-generated using specialist software by an independent trial administrator. Randomization will occur between the consent session and the first (baseline) home visit. After completing the consent session, the researcher will phone the trial administrator, who will consult the random number list to allocate the participant to a condition. A unique identification code denoting the participant and the participant’s general practice will be allocated by the trial administrator, to permit matching of data across time points.

### Study procedure

After consenting at the general practice, participants will be fitted with a waterproof accelerometer (activPAL3; PAL Technologies, Glasgow, Scotland) to wear continuously (day and night) for one week. Our preliminary work indicated that participants wished to complete questionnaires in their own time, and so a questionnaire of self-report behaviour, health and wellbeing measures will also be given at this point, for completion over the following week. One week later (that is, baseline), the patient will be visited at home and the accelerometer and questionnaire collected, objective measures taken and the intervention or control treatment administered. Further home visits will be conducted at 7 and 11 weeks post-baseline to fit patients with an accelerometer and give a further questionnaire, and at 8 and 12 weeks post-baseline, to collect accelerometers and questionnaires and obtain follow-up objective measures. Participants will be contacted by phone at 4 weeks to arrange the 7-week home visit. Each subsequent visit will be arranged at the previous visit. If a participant is not home for a scheduled visit, the visit will be rearranged by phone where possible. Where participants are not contactable by phone, a maximum of two letters (at 4-day intervals) will be sent to invite them to arrange follow-up home visits. Those who do not respond to the second letter will be treated as having dropped out of the study. Participants will receive a £10 shopping voucher at the baseline, 8-week and 12-week home visits, in recognition of their help in facilitating intervention assessment. They will also receive a further £30 shopping voucher at 12-week follow-up, conditional on having attended all home visits and returned the accelerometer.

Intervention and control treatments will be delivered to each participant individually by postgraduate or postdoctoral psychology researchers trained in describing the intervention and carrying out all necessary study procedures. Participants will be given and talked through the main messages of the appropriate booklet, and will have the opportunity to ask questions if they do not understand any part of the booklet. Participants will be asked to contact the lead researcher if they lose their booklet, to request that another free copy be sent by post. The active treatment period is 8 weeks.

### Intervention

For clarity, we report the content of our intervention with reference to the behaviour change technique (BCT) Taxonomy v1 [55]. The intervention booklet comprises text designed to promote motivation to form a habit, planning and self-monitoring to initiate and sustain change, and context-dependent repetition required for a habit to form [38]. The final intervention booklet will be made available on demand from the first author when the trial has ended.

Motivational text outlines the health detriments of prolonged sitting and the benefits of physical activity (BCT 5.1: ‘Information about health consequences’), and suggests reducing sedentary behaviour, either by directly substituting physical activity for existing sitting time (BCT 8.2: ‘Behaviour substitution’), or by promoting physical activity as a way of displacing sitting (BCT 4.1: ‘Instruction on how to perform behaviour’; BCT 13.2: ‘Framing/reframing’). The booklet explains that ‘physical activity’ refers not only to cardiovascular exercise, but also to stretching, balancing and muscle-strengthening exercises. It also describes and recommends the habit formation approach as a way of ingraining physical activity into daily routines, and provides guidance on how habits form (BCT 8.3: ‘Habit formation’).

Advice on forming habits is provided in the form of ten tips for physical activities that have the potential to displace sedentary behaviour and increase physical activity, and so improve health, wellbeing or functioning, and, if adopted and repeated consistently, lead to physical activity habit formation and disruption of sedentary behaviour habits (BCT 8.4: ‘Habit reversal’). The tips follow a template and feature catchy slogans to enhance memorability (for example, ‘take a stand’, ‘limber up’), brief descriptions of behaviour and the context in which it might best be done (BCT 1.4: ‘Action planning’), and, where appropriate, an explanation of how or why it will reduce sedentary behaviour or improve health or wellbeing. Additional ‘handy hints’ advise on how to move to higher-intensity variants of the recommended activity (BCT 8.7: ‘Graded tasks’), or ways to integrate the recommended activities into existing routines. Photos illustrate how to perform some tips (BCT 6.1: ‘Demonstration of behaviour’). The booklet is titled ‘On Your Feet to Earn Your Seat’, which reflects its recommendation that an empty seat be viewed as a cue to standing (BCT 7.1: ‘Prompts/cues’). Readers are encouraged to place the booklet on their favourite chair or on the kitchen table each evening to remind them to use it the following day.

Volitional support is provided in the form of a tick sheet with which participants can indicate whether they have achieved each of the tips at least once each day (BCT 2.3: ‘Self-monitoring of behaviour’; BCT 2.4: ‘Self-monitoring of outcomes of behaviour’), and advice on planning how to fit each recommendation into existing routines.

For those in the intervention group, the 4-week phone call to arrange the 7-week home visit will also provide an opportunity to discuss with a researcher any problems in adhering to the recommendations, or to obtain further motivational support (BCT 3.1: ‘Social support, unspecified’; BCT 3.2: ‘Social support, practical’).

### Control

Participants allocated to the control condition will receive an existing fact sheet outlining physical activity and sedentary behaviour recommendations for older adults [56]. This represents ‘usual care’ as a public health strategy for physical activity promotion among UK older adults. Since the intervention booklet details activities potentially beneficial to health in older adulthood, control participants will receive the intervention booklet on exiting the study.

### Measures

Table 1 outlines the study measurement schedule.

**Table 1:** **Study measurement schedule: behavioural, health and wellbeing measures**

Measurement	Number of items	Objective measure or self-report	Construct measured	Measurement points		
				Baseline	8 weeks	12 weeks
***Behavioural***						
Accelerometry	-	Objective	Physical activity, sedentary behaviour	x	x	x
International Physical Activity Questionnaire (short form) [51]	7	Self-report	Physical activity, sedentary behaviour	x	x	x
Sedentary behaviour questionnaire [52]	7	Self-report	Sedentary behaviour	x	x	x
Tick sheet *(intervention group only)*	10	Self-report	Adherence to intervention	*Continuous use between baseline and 8 weeks*		
***Psychological***						
Item from Self-Report Habit Index and Self-Report Behavioural Automaticity Index [64, 65]	2	Self-report	Generic physical activity habit, Generic sedentary behaviour habit	x	x	x
Item from Self-Report Habit Index and Self-Report Behavioural Automaticity Index [64, 65] *(intervention group only)*	10	Self-report	Habit for behaviours recommended in intervention booklet	x	x	x
CONFbal [59]	10	Self-report	Confidence in balance	x	x	x
Short Falls Efficiency Scale, International [60]	7	Self-report	Fear of falling	x	x	x
***Health and wellbeing***						
Item from Fall Risk Assessment Tool [58]	1	Self-report	History of falls	x	x	x
Items from Center of Epidemiological Studies Depression Scale [61]	2	Self-report	Fatigue, exhaustion	x	x	x
Verbal descriptor pain item [62]	1	Self-report	Pain	x	x	x
Verbal descriptor stiffness item	1	Self-report	Stiffness	x	x	x
EQ5D [57]	6	Self-report	Quality of life	x	x	x
Timed walk	-	Objective	Walking speed	x	x	x
Grip	-	Objective	Grip strength	x	x	x
Chair rises	-	Objective	Leg strength	x	x	x
Tandem standing	-	Objective	Balance	x	x	x
Blood pressure	-	Objective	Blood pressure	x	x	x
***Views towards intervention***						
Exit interview	-	Self-report	Acceptability of randomized controlled trial			x

#### Feasibility of study procedures

The time taken to complete measurements at each home visit will be recorded, to document the feasibility of study procedures. We will assess the feasibility of measuring outcomes listed in Table 1 and amend measures should participants experience difficulties in completing them. Recruitment and attrition rates will be recorded.

#### Demographics

Self-reported sex, age, postcode, ethnicity and education will be recorded.

#### Health, physical functioning and wellbeing

Physical health will be assessed objectively via blood pressure measurements, taken using an Omron M2 Classic monitor (Omron Healthcare, Kyoto, Japan), over the unclothed upper arm. Physical functioning will be objectively assessed via performance on a short battery of tests, including walking speed, hand grip strength (using a Jamar Hydraulic Hand Dynamometer; Patterson Medical, Warrenville, IL), chair rises and standing balance, which assess balance, gait, strength and endurance. Wellbeing will be self-reported using the EQ5D visual analogue scale [57]. Fall history in the year prior to baseline, and during the treatment period, will be self-reported using a single item derived from the Fall Risk Assessment Tool [58]. Confidence in balance will be self-reported using the CONFBal scale [59], and fear of falling will be self-reported using the international version of the Short Falls Efficacy Scale [60]. Fatigue will be self-reported using two items taken from the Center of Epidemiological Studies Depression Scale that assess the frequency with which, over the past week, ‘everything you did felt like an effort’ and ‘you could not get going’ [61]. Severity of general pain and stiffness over the previous week will each be self-reported using a single verbal descriptor item [62].

#### Behaviour measures

Among the intervention group, adherence to each of the activities recommended in the booklet will be self-reported on ‘tick sheets’ for eight weeks post-baseline. Participants will be requested to return tick sheets to the research team either by post in prepaid envelopes or at the 8-week follow-up visit.

Generic physical activity and sedentary behaviour will be self-reported using a short form of the International Physical Activity Questionnaire [51] and a validated sedentary behaviour questionnaire [52], and objectively measured over three 7-day periods using activPAL3 accelerometers (PAL Technologies, Glasgow, Scotland). These devices are lightweight, do not provide physical activity feedback to wearers, and are sensitive to transitions between sitting, lying down (both sedentary behaviours) and standing (a physical activity), so can distinguish true sedentary behaviour from inactivity [63]. Data on total sitting time, physical activity (step count, cadence (speed) of steps), and sit-to-stand transitions during each wear period will be entered into analyses.

Devices will be pre-programmed to begin data collection on the day following their fitting, and participants’ unique identifier codes will be programmed into each unit to facilitate data matching. The activPAL device will be fitted to the participant’s thigh, with the same thigh used for all wear periods, using a waterproof dressing to allow showering and bathing as normal.

#### Psychological measures

Habit strength for generic physical activity and sedentary behaviour and, in the intervention group only, each of the behaviours recommended in the tips will be assessed using a single automaticity item, adapted to each focal behaviour. This item (‘(Behaviour X) is something I do without thinking’) is derived from the automaticity subscale of the Self-Report Habit Index (SRHI) [64, 65]. A single item was chosen on the basis of data emerging from the preliminary field test that suggest that participants struggle to understand SRHI items, or show understandings that deviate from those intended by researchers. The chosen item has shown satisfactory content and predictive validity, and convergent validity with its parent index [64].

#### Experiences of the study

At the final measurement session, all participants will be interviewed about their experiences of the study. Topics will focus on convenience and acceptability of study procedures, potential for burden arising from participation, favourability towards the allocated leaflet, and potential behavioural and associated changes arising from the leaflet. This information will be used to document intervention acceptability and feasibility of study procedures, and, in the intervention group, experiences of habit formation.

### Data management, analysis and dissemination

#### Data management

All data will be generated and curated according to University College London procedures on data recording, storage and backup protocol. Data will be collected and entered into a database by the lead researcher (ITB). Only the lead researcher and chief investigator (BG) will have access to the data, which will be stored in a password-protected database on a secure shared network at University College London accessible to ITB and BG. To maintain confidentiality, personal data will be stored in a separate data file from all other study data. With the exception of consent forms, all personal data will be destroyed when data collection has been completed. Hand-completed consent forms and questionnaires will be stored in a locked filing cabinet in a locked office at University College London for five years after the trial ends, in line with institutional data storage policy.

#### Analysis of primary outcomes

The number of participants recruited in each month of the study, and the number attending each of the home visits, will be reported for each group. Differences in attrition rates between the two arms at each time-point will be explored using Chi-square tests, and reasons for drop-out, where available, will be reported.

#### Analysis of secondary outcomes

Analyses of changes in secondary outcomes (physical activity, habit, wellbeing, physical health, functioning) will be run using valid data at all three time points to analyze changes among completers only. Non-completers will be accounted for by using last-observation-carried-forward and baseline-observation-carried-forward analyses, the latter to ensure a more conservative assessment of potential changes in outcomes. Between-group changes in outcomes over the first 8 weeks will be assessed using *t* tests, and within-group changes examined using repeated measures analysis of variance (ANOVA). To evaluate whether habit change is associated with behaviour change, we will calculate correlations between changes in automaticity for generic physical activity and sedentary behaviour (and, in the intervention group, for each target habit), and changes in physical activity within each group. Effect sizes for changes in self-reported physical activity will be used to inform a power calculation to determine the sample size required for a definitive RCT. Qualitative interview data will be analyzed using thematic analysis.

#### Dissemination

Participants will be invited to request a brief summary of findings at the end of their involvement in the trial. Results will be disseminated to the public and policymakers via University College London and Age UK newsletters and websites. Results will be communicated to scientific audiences via presentations at relevant national and international conferences in behavioural science, geriatrics, physical activity, psychology and public health, and a manuscript submitted for publication in a journal covering one or more of these fields.

### Ethical issues

We believe that the risk posed to participants from the intervention and control treatments is minimal; the intervention is designed to promote everyday activities that may be easily absorbed into daily tasks, and the control booklet features information readily available via an NHS website. We deem the main ethical issue to be that of participant burden, and have attempted to mitigate this by offering shopping vouchers to recognise the participant’s contribution to the trial. The study has been approved by an NHS Research Ethics Committee (reference 13/LO/1549). Any amendments to the study procedures outlined will be submitted for approval to the research ethics committee as soon as they arise, and communicated to the scientific community in a planned paper reporting findings from the trial.

### Adverse events

We define an ‘adverse event’ as any untoward medical occurrence in a participant. All adverse events will be recorded and reported to the research ethics committee, where, in the opinion of the chief investigator, the event was related to the treatment and unexpected. Medical judgement will be exercised in deciding whether an adverse event is serious (that is, results in death, is life-threatening, requires hospitalizations or results in persistent or significant disability or incapacity).

### Trial management

Day-to-day management of the trial will be conducted by the lead researcher (ITB), who will meet fortnightly with the chief investigator (BG) to review progress. The research team (all authors of this paper) will meet once prior to the trial start date, once during the trial period and once following cessation of the trial.

An independent project steering committee has been assembled to oversee the wider intervention development project in which the current study is located, and will act as steering group for the current study. The committee comprises experts in health services research, sports and exercise science, clinical trials, and general practice, and includes a lay older adult representative. The committee has approved all study procedures described in this protocol, and will meet once during the trial period to review progress and audit trial conduct, and once following cessation of the trial to review results. A copy of the study findings will be submitted to the steering committee for approval prior to submission for dissemination. Given the exploratory nature of the trial, no independent data monitoring committee has been convened.

## Discussion

Older adults tend to spend too much time sitting and do too little physical activity [6]. Our study will assess the feasibility of evaluating an intervention for older adults that seeks to displace periods of prolonged sedentary behaviour with physical activity. Our intervention represents a ‘small changes’ approach, recommending forms of physical activity that can be performed at low intensity, and it uses principles of habit formation to promote the automatization and integration of these actions into everyday routines [37]. The intervention has been iteratively developed using empirical evidence of older adults’ activity preferences, verified as appropriate by a panel of experts. The intervention is theory-based, and the activities recommended in the tips are evidence-based, in that they have been shown to improve health and functioning outcomes. The RCT will compare our intervention booklet with a fact sheet that is readily available to older adults and that recommends physical activity and minimizing sedentary behaviour, but offers few suggestions of how this might be achieved. This exploratory RCT aims to test study procedures ahead of a future, fully-powered definitive RCT, but the collection of data on behaviour, habit, health, wellbeing at baseline and two follow-up points permits analysis of changes in these indices.

We acknowledge the limitations of our study design. Selection bias may impact findings. Enrolment into the study requires that patients return a declaration of interest by regular post, and attend their general practice to be consented. Older adults who leave the house more often tend to accrue more physical activity [54], and so these procedures favour those at the more active end of the inactivity spectrum, rather than the highly inactive and sedentary older adults who might benefit most from intervention. Taking consent at the practice was judged necessary to garner interest among practices, which receive financial incentives for consenting on the premises. We have attempted to minimize participant burden by conducting all post-recruitment procedures at participants’ homes, and offering shopping vouchers to recognise their efforts.

The intervention is designed to promote behaviour maintenance via habit formation, and it could be argued that the 12-week follow-up period is insufficient for maintenance to be observed. Habit strength has been shown to peak among individuals pursuing physical activity goals after an average of 90 days of daily repetition, six days longer than our follow-up [25]. However, the habit growth curve is typically asymptotic [25], and so notable gains in habit strength can be expected within our study time period [45]. While we cannot observe long-term behaviour maintenance, evaluation of the ‘Ten Top Tips’ intervention, on which our intervention is based, has shown that individuals who formed habits tended to exhibit greater weight loss at six-month follow-up [44].

A secondary aim of the trial is to conduct tests of effects on behavioural and health outcomes, but our two-arm design makes it difficult to identify effects or sources of effects. Should no between-group differences be found, it will be unclear whether true intervention effects have been suppressed because the control treatment has a similar impact to the intervention, or because neither is effective. Should the intervention show greater impact on secondary outcomes than the control, we will be unable to reliably identify the ‘active ingredients’ of the intervention. Although we will estimate the contribution of habit formation by assessing correlations between gains in habit and behaviour, this analysis will not definitively establish whether habit formation is responsible for behaviour change. Habit formation has been documented in trials of interventions that neither explicitly promoted context-dependent repetition, nor explained the habit formation process to participants [66, 67]. A third arm, with a matched non-habit-based version of our intervention leaflet, is needed to isolate effects of habit-based advice [23].

The primary aim of this study is to assess the feasibility of trialling our intervention. Should the trial appear feasible, and the intervention leaflet show promise in modifying sitting and physical activity, a definitive large-scale trial conducted over a longer-term period with multiple arms will help to shed light on effects and their likely causes. At this stage in the intervention development process, a pragmatic RCT offers the most logical first step.

## Trial status

The proposed trial has not yet begun.

## Endnote

^a^This study is listed on the ISRCTN Register, and has been approved by a research ethics committee, under the title ‘Increasing physical activity in older adults’. The title deviates from that of this manuscript because our preliminary work suggested that the target population better understands the term ‘physical activity’ than ‘sedentary behaviour’. We do not view this discrepancy as problematic because, given that standing is a form of physical activity, reducing sedentary behaviour necessarily involves increasing physical activity.

## Authors’ information

BJJ is funded by a National Institute for Health Research Postdoctoral Fellowship (2010-03-023). MH is supported by the British Heart Foundation (RE/10/005/28296).

## Electronic supplementary material

Additional file 1: **SPIRIT checklist.** (DOCX 154 KB)

Additional file 2: **TIDieR checklist.** (DOCX 34 KB)

Additional file 3: Figure S1: Preliminary work: flow diagram of sources and methods for generating and field testing intervention content. (DOCX 68 KB)

## Abbreviations

ANOVA: analysis of variance
BCT: behaviour change technique
ISRCTN: International Standard RCT Number Register
NHS: UK National Health Service
RCT: randomized controlled trial
SPIRIT: Standard Protocol Items: Recommendations for Interventional Trials
SRHI: Self-Report Habit Index
TIDieR: Template for Intervention Description and Replication.
