# Supplementary material for: ‘On Your Feet to Earn Your Seat’, a habit-based intervention to reduce sedentary behaviour in older adults: study protocol for a randomized controlled trial
Source: Trials. 2014 Sep 20;15:368. doi: 10.1186/1745-6215-15-368 (PMC4180306; doi:10.1186/1745-6215-15-368)
Supplement: Supplementary file 3 — Additional file 3: Figure S1: Preliminary work: flow diagram of sources and methods for generating and field testing intervention content. (DOCX 68 KB) [file 13063_2014_2237_MOESM3_ESM.docx]

**Supplementary figure.** Preliminary work: Flow diagram of sources and methods for generating and field-testing intervention content

| *Generation of intervention content* | | | | *Field-testing intervention content* |
| --- | --- | --- | --- | --- |
| Research team | Older adult group 1  (n = 17) | Expert panel  (n = 15) | Older adult group 2  (n = 23) | Older adult group 3  (n = 25) |

1. Focus groups (n = 10) & interviews (n = 17)

8. Fourth iteration of tips & motivational text, graphic design of booklet

6. Third iteration of tips & motivational text

4. Second iteration of tips & motivational text

2. First iteration of tips & motivational text

3. Feedback on first iteration of tips

5. Focus groups (n = 6) & interviews (n = 11)

7. Feasibility ratings

9. Pre-post acceptability field-test
